# Mesenchymal Stem Cell: A Friend or Foe in Anti-Tumor Immunity

**DOI:** 10.3390/ijms222212429

**Published:** 2021-11-18

**Authors:** Carl Randall Harrell, Ana Volarevic, Valentin G. Djonov, Nemanja Jovicic, Vladislav Volarevic

**Affiliations:** 1 Regenerative Processing Plant, LLC, 34176 US Highway 19 N, Palm Harbor, FL 34684, USA; dr.harrell@regenerativeplant.org; 2 Department of Cognitive Psychology, Faculty of Medical Sciences, University of Kragujevac, 69 Svetozar Markovic Street, 34000 Kragujevac, Serbia; ana.volarevic@medf.kg.ac.rs; 3 Institute of Anatomy, University of Bern, Baltzerstrasse 2, 3012 Bern, Switzerland; valentin.djonov@ana.unibe.ch; 4 Department of Histology and Embryology, Faculty of Medical Sciences, University of Kragujevac, 69 Svetozar Markovic Street, 34000 Kragujevac, Serbia; nemanjajovicic.kg@gmail.com; 5 Department of Genetics, Faculty of Medical Sciences, University of Kragujevac, 69 Svetozar Markovic Street, 34000 Kragujevac, Serbia; 6 Department of Microbiology and Immunology, Faculty of Medical Sciences, University of Kragujevac, 69 Svetozar Markovic Street, 34000 Kragujevac, Serbia

**Keywords:** mesenchymal stem cells, regulation, immune response, tumor, immunotherapy

## Abstract

Mesenchymal stem cells (MSCs) are self-renewable, multipotent stem cells that regulate the phenotype and function of all immune cells that participate in anti-tumor immunity. MSCs modulate the antigen-presenting properties of dendritic cells, affect chemokine and cytokine production in macrophages and CD4+ T helper cells, alter the cytotoxicity of CD8+ T lymphocytes and natural killer cells and regulate the generation and expansion of myeloid-derived suppressor cells and T regulatory cells. As plastic cells, MSCs adopt their phenotype and function according to the cytokine profile of neighboring tumor-infiltrated immune cells. Depending on the tumor microenvironment to which they are exposed, MSCs may obtain pro- and anti-tumorigenic phenotypes and may enhance or suppress tumor growth. Due to their tumor-homing properties, MSCs and their exosomes may be used as vehicles for delivering anti-tumorigenic agents in tumor cells, attenuating their viability and invasive characteristics. Since many factors affect the phenotype and function of MSCs in the tumor microenvironment, a better understanding of signaling pathways that regulate the cross-talk between MSCs, immune cells and tumor cells will pave the way for the clinical use of MSCs in cancer immunotherapy. In this review article, we summarize current knowledge on the molecular and cellular mechanisms that are responsible for the MSC-dependent modulation of the anti-tumor immune response and we discuss different insights regarding therapeutic potential of MSCs in the therapy of malignant diseases.

## 1. Introduction

The term “anti-tumor immunity” refers to the innate and adaptive immune response elicited against tumor antigens [1]. Critical aspects in the interaction between tumor and immune cells are the generation, expression and release of tumor antigens and their consequent engulfment and processing by dendritic cells (DCs) [2]. DCs capture tumor antigens and present them within major histocompatibility class (MHC) molecules to the naïve CD4+ and CD8+ T lymphocytes, resulting in their activation, proliferation and differentiation in effector CD4+ T helper (Th)cells and CD8+ cytotoxic T lymphocytes (CTLs) [2]. CD4+Th cells orchestrate the anti-tumor immune response through the production of interleukin (IL)-2, which increases the proliferation of CD8+ CTLs and through the secretion of interferon gamma (IFN-γ), which induces the generation of the anti-tumorigenic M1 phenotype in tumor-infiltrated macrophages (TAMs) [1,3]. M1 macrophage-derived chitinases and proteases lyse tumor cells while M1 macrophage-sourced chemokines attract cytotoxic CD8+ CTLs and natural killer (NK) cells in the tumor microenvironment [1,3]. CTLs and NK cells share common effector mechanisms for the elimination of cancer cells: granule exocytosis and the death ligand/death receptor system [4,5]. CTLs/NK cell-sourced perforin forms pores in the membranes of tumor cells, allowing granzyme B to access cytosol where it induces the apoptosis of cancer cells by cleaving critical intracellular substrates, which control their survival [4]. CTLs and NK cells express cell death ligands (program death ligand (PDL) and Fas ligand (FASL)), which activate extrinsic and intrinsic mitochondrial apoptotic pathways in malignant cells through the binding to program death (PD) and Fas receptors that are expressed on their membranes [1,4,5]. In contrast to M1 macrophages, CTLs and NK cells, immunosuppressive CD4+FoxP3+Tregulatory cells (Tregs), tumor-associated M2 macrophages, N2 neutrophils and myeloid-derived suppressor cells (MDSCs) promote tumor growth and progression [6]. Tregs and MDSCs express cytotoxic T lymphocyte-associated protein 4 (CTLA-4) and PDL1 and produce immunosuppressive cytokines (IL-10 and transforming growth factor beta (TGF-β)) that inhibit the proliferation, activation and effector function of CTLs and NK cells, while M2 macrophages and N2 neutrophils secrete pro-angiogenic factors (vascular endothelial growth factor (VEGF), TGF-β, prostaglandin E2 (PGE2)), which induce the generation of new blood vessels, enabling enhanced tumor growth and progression [1,3,6]. Since different immune cells affect tumor growth in opposite directions, the modulation of their phenotype and function represents a potentially new therapeutic approach in cancer treatment.

Mesenchymal stem cells (MSCs) are self-renewable, multipotent stem cells that regulate innate and adaptive immune response in almost all adult tissues [7]. After injury, alarmins, released from damaged cells, activate tissue-resident MSCs, which express PDL1 and produce various numbers of immunoregulatory factors that modulate the cytokine milieu of local microenvironments, altering the phenotype and function of immune cells [8]. MSCs affect the antigen-presenting properties of DCs, B cells and macrophages, modulate the phagocytic ability of neutrophils and monocytes, change the polarization of macrophages, modify the xcytotoxic properties of NK cells and regulate the proliferation, activation and effector functions of CD4+ and CD8+ T cells [7,8]. Additionally, MSCs, in a juxtacrine and paracrine manner, induce the generation and expansion of immunosuppressive Tregs and MDSCs, playing a crucially important role in the alleviation of on-going inflammation [7,8].

Since MSCs represent an important cellular constituent of the tumor microenvironment and are able to modulate the phenotype and function of all immune cells that participate in the anti-tumor immune response [9,10], a large number of experimental studies investigated the molecular mechanisms that were responsible for the MSC-based modulation of anti-tumor immunity. Their findings, obtained in different animal models, suggested that MSCs could either support or suppress tumor progression, since many factors affected MSC-dependent immunomodulatory properties in the tumor microenvironment [9,10,11,12,13]. In this review article, we delineate signaling pathways that regulate the cross-talk between MSCs, immune cells and tumor cells, describe in detail the molecular and cellular mechanisms that were responsible for the MSC-dependent modulation of anti-tumor immune response and discuss different insights regarding the therapeutic potential of MSCs in the therapy of malignant diseases. An extensive literature review was carried out in September 2021 across several databases (MEDLINE, EMBASE, Google Scholar, ClinicalTrials.gov). Keywords used in the selection were: “mesenchymal stem cells”, “immunomodulation”, “tumor cells”, ”immune cells”, “dendritic cells”, “macrophages”,” “T cells”, “NK cells”, “malignant diseases“, and “cell-based therapy”. All journals were considered, and an initial search retrieved 2538 articles. The abstracts of all these articles were subsequently reviewed by two of the authors (CRH and VV) to check their relevance to the subject of this manuscript. Eligible studies were required to delineate the effects of MSCs on the phenotype and function of tumor-infiltrated immune cells, and their findings are analyzed in this review.

## 2. MSC-Dependent Suppression of Anti-Tumor Immunity

**Figure 1:**
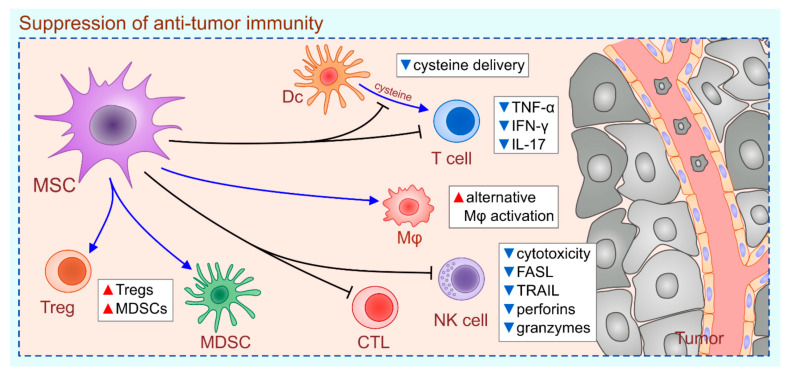
MSC-based suppression of anti-tumor immunity. MSCs promote tumor growth by: (i) preventing DC-based cysteine delivery to T cells, (ii) inducing alternative activation of tumor-associated macrophages, (iii) suppressing production of TNF-α, IFN-γ and IL-17 in CD4+T helper cells, (iv) inhibiting cytotoxicity of FASL on CTLs and NK cells (down-regulated expression of FASL and TRAIL; reduced secretion of perforins and granzymes) and (v) promoting generation and expansion of Tregs and MDSCs. The red triangles represent increased number of cells and their products; the blue triangles represent the decreased number of cells and their products.

Several lines of evidence demonstrated that both tumor-resident (cancer-associated MSCs (CA-MSCs)) and exogenously administered MSCs promoted tumor growth by: (i) preventing the DC-dependent activation of naive T cells, (ii) inducing the alternative activation of TAMs, (iii) modulating cytokine production in T helper cells, (iv) down-regulating the cytotoxicity of CTLs and NK cells and (v) promoting the generation and expansion of Tregs and MDSCs (Figure 1) [14,15,16,17,18,19,20,21].

Ghosh and colleagues demonstrated that CA-MSCs in a paracrine, IL-10 and STAT-3-dependent manner suppressed the DC-dependent activation of naïve T cells [14]. CA-MSC-derived IL-10 inhibited the DC-induced proliferation of T cells by blocking the ability of DC to provide cysteine to cognate T lymphocytes. T cells lack cystathionase, and therefore could not synthesize cysteine from their intracellular stores [15]. Accordingly, T cells use their plasma membrane neutral amino acid transporter to import cysteine released from DCs [15]. CA-MSC-derived IL-10 induced the phosphorylation of STAT-3 in DCs [14]. Phosphorylated STAT-3 entered the nucleus and repressed the interferon gamma-activated sequence (GAS) which served as a cystathionase promoter sequence [14]. As a consequence, DC-derived cysteine export to T cells is suppressed, resulting in reduced T cell proliferation and activation. Lack of cysteine significantly attenuated the production of IFN-γ in T cells and alleviated their capacity to activate macrophages in an IFN-γ-dependent manner [14].

The cross-talk between MSCs, anti-tumorigenic (M1) and pro-tumorigenic (M2) macrophages is crucially responsible for the MSC-dependent regulation of tumor progression [12]. In order to demonstrate the effects of M1 macrophages on the MSC-dependent modulation of tumor growth, Jia and colleagues treated MSCs with condition medium derived from TNFα, IL-1β, and iNOS-expressing M1 macrophages (MSC^M1-CM^) and compared their phenotype and function with BM-MSCs that grew under standard conditions (MSC^control^) [16]. The MSC^M1-CM^ displayed greater potential to promote tumor growth in comparison with MSC^control^. MSC^M1-CM^, but not MSC^control^, remarkably increased the tumor-initiating ability and tumor growth of MDA-MB-231-FLUC breast cancer cells [16]. Similarly, MSC^M1-CM^ showed a better tumor-promoting effect than MSC^control^ in the murine models of hepatocellular carcinoma and glioblastoma [16]. The M1 macrophage-derived secretome increased the expression of toll-like receptor 3 (TLR-3) on MSCs. TLR-3 signaling promoted the generation of the immunosuppressive MSC2 phenotype in MSCs by increasing the expression of inducible nitric oxide synthase (iNOS), CCL2, IL-6 and cyclooxygenase 2 (COX-2) [16]. MSC^M1-CM^ suppressed the production of activated T cells in an iNOS and nitric oxide (NO)-dependent manner [16]. When MSC^M1-CM^ were exposed to siRNA that inhibited iNOS activity and NO production, the immunosuppressive properties of MSC^M1-CM^ were significantly down-regulated. The tumor-promoting activity of the MSCs in vivo was largely dependent on their capacity for enhanced production of CCL2, COX-2 and IL-6. MSC^M1-CM,^ in a CCL2-dependent manner, elicited the accumulation of CCR2-expressing M1 macrophages in tumor tissue [16]. M1 macrophages, in turn, in a TNF-α-dependent manner induced the generation of the immunosuppressive MSC2 phenotype in MSCs. MSC2 had an increased capacity for the production of IL-6 and COX-2, which induced the generation of the anti-inflammatory and pro-tumorigenic M2 phenotype in TAMs. M2 macrophages, through increased production of immunosuppressive cytokines (IL-10 and TGF-β) and pro-angiogenic factors (VEGF, PGE2), enabled enhanced tumor growth and progression [16].

The cross-talk between CA-MSCs and tumor-infiltrating M2 macrophages was the research focus of Mathew and colleagues [17], who demonstrated that CA-MSCs promoted the growth of pancreatic cancer by inducing the M2 polarization of TAMs. Compared to bone-marrow-derived MSCs (BM-MSCs), CA-MSCs had higher capacity for the production of immunosuppressive cytokines IL-10 and TGF-β, growth factors (monocytes colony-stimulating factor (*M-CSF),* granulocyte–macrophage colony-stimulating factor (*GM-CSF)* and chemokine (CCL2) and had increased tumor-promoting ability [17]. Significantly enhanced growth and progression of pancreatic cancer was noticed in CA-MSC-treated mice compared to BM-MSC-treated animals [17]. CA-MSC-sourced IL-6 and IL-10 induced the generation of M2 TAMs in pancreatic tissue, while CA-MSC-derived CCL2 was mainly responsible for the increased influx of circulating M2 monocytes into the pancreatic tumors. An increased presence of M2 TAMs was crucially responsible for optimal tumor-promoting activity of CA-MSCs, since their depletion significantly reduced the tumor growth of CA-MSC-treated mice [17]. M2 TAMs produced IL-10 and IL-1 receptor antagonist (IL-1Ra) which enabled the generation of the MSC2 phenotype in MSCs. Immunosuppressive cytokines produced by M2 TAMs and MSC2 down-regulated the anti-tumor immune response, enabling immune evasion and the uncontrolled proliferation of pancreatic cancer cells [17].

An M2 TAM-generated anti-inflammatory tumor microenvironment was crucially responsible for the MSC-dependent suppression of tumor-infiltrated CD8+ CTLs [18]. Hypoxia and inflammation, generated during tumor progression, induce the massive release of nucleotides (ATP and ADP) from dead parenchymal cells [18]. MSC2 expresses CD39 and CD73 ectonucleotidases, which hydrolase ATP and ADP and generate high levels of adenosine in the tumor microenvironment. Adenosine exerts immunosuppressive effects on CD8+ CTLs by binding to adenosine-specific receptor A2A [18]. MSC-based activation of the adenosine:A2A axis in CTLs resulted in the enhanced generation of cAMP, which suppressed the proliferation of CTLs, attenuated the production of anti-tumor cytokines (TNF-α and IFN-γ) and inhibited the release of perforins and granzyme B in CTLs [18]. In line with these findings are results recently obtained by Liu and colleagues, who showed that the BM-MSC-dependent suppression of the anti-tumor properties of CTLs was crucially responsible for the progression of multiple myeloma (MM) [19]. Through the activation of the PDL1/PD1 axis, PDL1-expressing BM-MSCs induced apoptosis and inhibited the exocytosis of perforin and granzyme B in CTLs of MM patients. Accordingly, the use of PDL1 inhibitor, which inhibited the BM-MSC-based suppression of CTLs and enhanced the CTL-dependent elimination of tumor cells, had beneficial effects in the treatment of MM patients [19].

In addition to CTLs, CA-MSCs also regulate the phenotype, function and cytotoxic properties of tumor-infiltrated NK cells [20]. The cross-talk between CA-MSCs and NK cells is crucially important for the optimal MSC-driven suppression of anti-tumor immunity [20]. NK cells may recognize one or many molecules expressed on CA-MSCs, including MHC class I polypeptide-related sequence (MICA), UL16 binding proteins (ULBPs), CD112 and CD155, which serve as ligands for NK cell-activating receptors [21]. Activated NK cells either induce apoptosis or, through the increased production of IFN-γ, induce the generation of the immunosuppressive MSC2 phenotype in neighboring CA-MSCs. In turn, CA-MSC2 regulate the proliferation, cytotoxicity and cytokine production of tumor-infiltrating NK cells. MSCs in a juxtacrine, contact-dependent manner down-regulated the expression of cytotoxic receptors (NKp44, NKp30, NKG2D, DNAM-I) on CD56^dim^NK cells, while in a paracrine, PGE2-dependent manner suppressed IFN-γ production in CD56^brigh^ NK cells [21].

CA-MSCs induce the generation and expansion of MDSCs and Tregs that attenuate anti-tumor immunity and support tumor growth and progression [22,23,24,25,26,27]. MDSCs produce a large number of immunosuppressive molecules (Arginase-1, NO, TGF-β, IL-10) that inhibit the proliferation and activation of naïve T cells, induce apoptosis and promote the G0/G1 cell cycle arrest of effector Th1 and Th17 cells, attenuate the cytotoxicity of CTLs and NK cells, induce the alternative activation of TAMs and promote the expansion of Tregs [22]. IFN-γ, mainly derived from tumor-infiltrating Th1 lymphocytes and NK cells, is crucially important for the generation and immunosuppressive function of MDSCs [23]. IFN-γ induces the enhanced expression of immunoregulatory molecules (PDL1 and CD40) on MDSCs and increases the synthesis of PGE2 and S100A8/A9, which, in an autocrine manner, induce the proliferation and activation of MDSCs [23]. MSCs promote proliferation and inhibit the apoptosis of MDSCs [24]. Additionally, MSCs enhance the immunosuppressive properties of MDSCs by increasing the production of NO and TGF-β. Consequently, MSC-primed MDSCs had increased capacity for the suppression of T cell-driven anti-tumor immunity in vivo [24].

Tregs express immunoregulatory molecules (PDL1 and CTLA4) and produce immunosuppressive cytokines (IL-10, IL-35, TGF-β), which inhibit the synthesis of TNF-α, IFN-γ and IL-17 in Th1 and Th17 cells and reduce the production of perforin and granzymes in CTLs, alleviating their anti-tumor properties [25]. MSCs in an indoleamine 2,3-dioxygenase (IDO)-dependent manner induce the generation and expansion of Tregs [26,27]. MSC-sourced IDO is a heme-containing enzyme that converts tryptophan (TRP) to immunosuppressive kynurenine (KYN) [7]. KYN promotes the expression of Treg lineage-defining transcription factor (forkhead box P3, FoxP3) in naïve T cells, enabling the generation of immunosuppressive CD4+FoxP3+Tregs in lymph organs [7]. Additionally, in the tumor microenvironment, MSC-sourced IDO prevents the trans-differentiation of Tregs in anti-tumorigenic, Th17-like cells [26]. Protein kinase B (PKB) and mammalian target of rapamycin (mTOR) are elicited by the binding of tumor antigens to the T cell receptor (TCR) of Tregs. Activated PKB and mTOR induce the generation of a pro-inflammatory and anti-tumorigenic phenotype in Tregs by enhancing the production of TNF-α, IL-17 and IL-22. A low level of TRP in the tumor microenvironment activates the general control non-derepressible 2 (GCN2) kinase, which prevents the phosphorylation of PKB and inhibits PKB/mTOR signaling. By converting TRP to KYN, MSCs-sourced IDO induces low TRP levels, activates GCN2 kinase and suppresses PKB/mTOR signaling in tumor-infiltrating Tregs, preventing their trans-differentiation in anti-tumorigenic Th17-like cells [7].

CA-MSCs also induced the generation of a regulatory phenotype in B cells [28]. Significantly attenuated production of TNF-α and increased production of IL-10 were noticed in CA-MSC-primed B cells. In vivo, the CA-MSC-dependent induction of the regulatory phenotype in B cells contributed to the creation of systemic immunosuppression that enabled enhanced tumor growth and progression [28].

Exogenously administered MSCs use the same molecular and cellular mechanisms as CA-MSCs to suppress anti-tumor immunity [29]. By using a murine metastatic model of lung cancer, we demonstrated that intravenously injected BM-MSCs significantly augmented lung cancer metastasis by down-regulating the anti-tumor immune response [29]. MSCs suppressed the production of TNF-α in DCs and macrophages and induced the polarization of TNF-*α*-producing CD4+ Th1 and IL-17-producing Th17 cells in IL-10-producing Tregs. Accordingly, serum levels of anti-tumorigenic cytokines (TNF-α and IL-17) were decreased and the serum concentration of immunosuppressive IL-10 was increased in MSC-treated tumor-bearing animals. MSCs suppressed the cytotoxicity of CTLs and NK cells in metastatic lungs by down-regulating the expression of FasL and NKG2D and by reducing the exocytosis of perforins and granzymes. MSC-sourced IDO and NO were mainly responsible for the pro-tumorigenic effects of MSCs, since the pharmacological inhibition of IDO and iNOS activity completely abrogated the MSC-driven suppression of anti-tumor immunity in tumor-bearing mice [29].

## 3. Immunoregulatory Effects of Exogenously Injected MSCs Depend on the Time of Their Administration

Within the tumor microenvironment, MSCs are constantly exposed to the growth factors and cytokines released by tumor-infiltrating immune cells, endothelial cells and tumor cells [9]. Despite the fact that a large number of studies demonstrated the pro-tumorigenic potential of MSCs, it has to be noted that MSCs are not constitutively immunosuppressive cells [12]. In fact, MSCs are a double-edged sword in relation to anti-tumor immunity [12]. As plastic cells, MSCs may adopt phenotype and function under the influence of the biological factors to which they are exposed [30]. MSCs may obtain pro-inflammatory (MSC1) and anti-inflammatory (MSC2) phenotypes depending on the local tissue concentration of inflammatory cytokines, TNF-α and IFN-γ. When MSCs engraft in the tissue with a low level of TNF-α and IFN-γ, they obtain a pro-inflammatory MSC1 phenotype, secrete a large number of inflammatory factors (reactive oxygen species (ROS) IL-1β, interferon alpha and beta (IFN-α, IFN-β), TNF-α and IFN-γ), which enhance the phagocytic properties of neutrophils and macrophages and the cytotoxicity of CTLs and NK cells. In contrast, when MSCs are exposed to high levels of inflammatory cytokines (TNF-α and IFN-γ), they acquire an immunosuppressive MSC2 phenotype characterized by the increased production of anti-inflammatory factors (TGF-β, IL-10, PGE2, NO, IDO, IL-1Ra) that suppress the effector function of inflammatory immune cells and attenuate on-going inflammation. Additionally, TNF-α and IFN-γ-primed MSC2 express and secrete PDL1 and PDL2, which suppress the proliferation of TNF-α and IFN-γ-producing T cells and promote the generation and expansion of immunosuppressive Tregs [31].

In line with these findings, we recently observed that the effects of exogenously administered MSCs on anti-tumor immunity and tumor progression depended on the time of their inoculation in tumor-bearing animals [32]. MSCs transplanted during the initial phase of melanoma growth exerted a tumor-suppressive effect, while MSCs injected during the progressive stage of melanoma development suppressed antitumor immunity and enhanced tumor expansion. MSCs intravenously injected 24 h after melanoma induction significantly enhanced the cytotoxicity of CD8+ CTLs and NK cells, increased the production of anti-tumorigenic cytokines (TNF-α, IFN-γ, IL-17) in tumor-infiltrated CD4+ Th1 and Th17 lymphocytes and attenuated melanoma growth and progression. Contrasting findings were observed in melanoma-bearing mice that intravenously received MSCs 14 days after tumor induction [32]. Enhanced tumor growth, loss of weight and decreased survival were consequences of the MSC-induced suppression of anti-tumor immunity. MSCs significantly reduced the total number of tumor-infiltrated, MHC class II and CD80-expressing, IL-12-producing DCs and TAMs and attenuated their antigen-presenting properties [32]. Additionally, MSCs down-regulated the secretion of perforine and granzyme B-containing vesicles from activated CTLs and NK cells, alleviating their tumoricidal potential. Moreover, MSCs injected 14 days after tumor induction induced the generation of an immunosuppressive phenotype in CD4+T lymphocytes and prevented the trans-differentiation of TGF-β and IL-10-producing Tregs into anti-tumorigenic IFN-*γ*- and IL-17-producing Th1 and Th17 cells [32]. Since low levels of inflammatory cytokines (TNF-α, IFN-γ) were measured in plasma samples of tumor-bearing mice 24 h after tumor induction and concentrations of these inflammatory cytokines increased during tumor progression, we assume that MSCs that were injected during the initial phase of melanoma development engrafted in a “pro-MSC1 tumor microenvironment” and obtained an anti-tumorigenic MSC1 phenotype, while MSCs that were administered during the progressive stage of melanoma progression engrafted in a “pro-MSC2 tumor microenvironment” and consequently acquired an immunosuppressive and pro-tumorigenic MSC2 phenotype [32].

## 4. MSCs as Potentially New Therapeutic Agents in Immunotherapy of Cancer

MSCs do not express MHC class II molecules and therefore could be transplanted in MHC-mismatched recipients [33]. Additionally, MSCs express various chemokine receptors and after intravenous injection migrate to the tumor tissue to participate in the anti-tumor immune response [33]. Accordingly, several clinical studies evaluated the anti-tumor properties of MSCs (Table 1).

Because of their low immunogenicity and tumor-homing properties, MSCs have been explored as vehicles for the delivery of bi-specific T cell-engaging antibodies, protein engagers that simultaneously bind to the tumor antigen and appropriate ligand on T lymphocytes, enabling the specific T cell-mediated elimination of tumor cells [34]. Glypican 3 (GPC3) is a protein that regulates the proliferation of hepatocellular carcinoma cells [35]. Szoor and colleagues used MSCs that expressed GPC3-specific single-chain variable fragment (scFv) and CD3-specific scFv (MSC^GPC3-CD3^) to direct GPC3-specific CD4+ T helper cells and CD8+ CTLs towards the GPC3-expressing hepatocellular carcinoma cells [34]. Co-culture of GPC3+ tumor cells, MSCs^GPC3-CD3^ and T lymphocytes led to the increased production of IFN-γ in GPC3-specific CD4+T cells and the enhanced activation and expansion of GPC3-specific CTLs, which resulted in the efficient CTL-dependent killing of GPC3-expressing malignant cells. Similar findings were observed in vivo. An increased activation of GPC3-specific T cells and significantly reduced hepatocellular carcinoma growth were observed in MSCs^GPC3-CD3^-treated tumor-bearing mice, demonstrating the therapeutic potential of MSCs^GPC3-CD3^ in the immunotherapy of hepatocellular carcinoma [34].

Low doses of ultraviolet radiation and X-ray irradiation generate an anti-tumorigenic MSC1 phenotype in MSCs, and therefore may be used for MSC priming [36]. Irradiated BM-MSC1 secretes a large amount of TNF-*α* and IFN-γ, which: (i) inhibit the proliferation of tumor cells by deregulating Wnt and TGF-*β*/Smad signaling and (ii) induce the apoptosis of tumor cells by blocking their cell cycle in the G1 phase. Additionally, MSC-sourced TNF-*α* induces the necrosis of tumor cells and enhances the expression of E and P selectins on tumor endothelial cells, enabling a massive influx of immune cells [36]. MSC-sourced IFN-*γ* induces the generation of the anti-tumorigenic (M1) phenotype in TAMs and enhances the cytotoxicity of tumor-infiltrated CTLs and NK cells. Upon activation by MSC-derived IFN-γ, CD8+ CTLs and NK cells up-regulate the expression of FASL and TRAIL and increase the release of perforin and granzymes that induce the apoptosis of tumor cells [36]. IFN-γ-primed M1 macrophages either phagocyte apoptotic tumor cells or secrete ROS, NO and *TNF-*α, which have direct cytotoxic effects on malignant cells [36].

**Figure 2:**
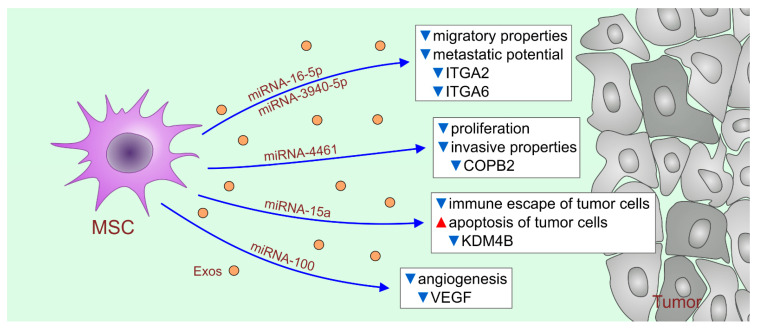
MSC-EVs as potentially new therapeutic agents in immunotherapy of malignant diseases. MSC-EVs deliver anti-tumorigenic miRNAs directly into tumor cells, altering their viability, proliferation rate and invasive characteristics. MSC-EV-sourced miRNA-16-5p and miRNA-3940-5p inhibited migratory properties and metastatic potential of tumor cells by down-regulating expression of integrins ITGA2 and ITGA6 on their membranes. MSC-EV-delivered miRNA-4461 suppressed proliferation and invasive properties of tumor cells by reducing expression of COPB2, which is essential for Golgi budding and vesicular trafficking. MSC-EVs carrying miRNA-15a inhibit the immune escape of tumor cells by regulating expression of HOXC4, which binds to the promoter sequence of PDL1, controlling its synthesis and membrane expression. MSC-derived miRNA-15a induces apoptosis of tumor cells by inhibiting activity of KDM4B which epigenetically regulates chromatin structure. The MSC-EV-delivered miRNA-100 down-regulates production of VEGF in cancer cells, preventing generation of new blood vessels in growing tumors. The red triangle represents increased and the blue triangles represent decreased activity.

Several lines of evidence demonstrated that the administration of MSC-derived extracellular vesicles (MSC-EVs), which contain MSC-sourced anti-tumorigenic microRNAs (miRNAs), could represent a potentially new therapeutic approach in the MSC-based immunotherapy of tumors [10,13,37]. Due to the lipid envelope, MSC-EVs easily by-pass all biological barriers and deliver their cargo directly into the target cells [38]. Accordingly, MSC-EVs could deliver MSC-sourced anti-tumorigenic miRNAs directly into tumor cells, altering their viability, proliferation rate and invasive characteristics (Figure 2) [10,13]. Human BM-MSC-EV-sourced miRNA-16-5p and miRNA-3940-5p from human umbilical cord-derived MSC-EVs (UC-MSC-EVs) inhibited the migratory properties and metastatic potential of tumor cells by down-regulating the expression of Integrin Subunit Alpha (ITGA)2 and ITGA6 on their membranes [39,40]. Human BM-MSC-EV-delivered miRNA-4461 suppressed the proliferation and invasive properties of tumor cells by reducing the expression of COPB2, which is essential for Golgi budding and vesicular trafficking [41]. Human adipose tissue-derived MSC-EVs (AT-MSC-EVs) carrying miRNA-15a inhibit the immune escape of tumor cells by regulating the expression of homeobox C4 (HOXC4), which binds to the promoter sequence of PDL1, controlling its synthesis and membrane expression [42]. Additionally, human AT-MSC-EV-derived miRNA-15a induces the apoptosis of tumor cells by inhibiting the activity of histone lysine demethylase 4B (KDM4B), which epigenetically regulates chromatin structure [42]. Human BM-MSC-EV-delivered miRNA-100 down-regulates the production of VEGF in cancer cells, preventing the generation of new blood vessels in growing tumors [43].

Because of their high affinity to the tumor tissue and increased resistance to the majority of chemotherapeutic drugs, MSCs have been explored as targeted delivery agents of anti-cancer drugs [44]. Reduced numbers of lung metastases were noticed in melanoma-bearing animals that received MSCs loaded with the anti-cancer drug paclitaxel (PTX) [44]. Similar results were reported by Layek and colleagues who demonstrated enhanced anti-tumor properties of PTX-loaded nano- and glyco-engineered MSCs against murine and ovarian cancer [45,46,47]. As vehicles, MSCs had many advantages in comparison to other drug administration agents [44]. Anti-neoplastic drug-loaded MSCs released chemotherapeutics directly in the site of primary and metastatic tumors without affecting neighboring tissues [44]. Accordingly, reduced side effects, an increased half-life and better anti-tumor effects were noticed in experimental animals that received anti-cancer drug-loaded MSCs compared to chemotherapeutic-treated tumor-bearing animals [44].

## 5. Conclusions

Genetically engineered MSCs that express bi-specific T cell-engaging antibodies and produce anti-tumorigenic miRNAs could be used as new therapeutic agents in the immunotherapy of malignant diseases. Bearing in mind that MSCs may alter their phenotype and function in the tumor microenvironment, future experimental studies should be focused on the long-term follow-up of MSC-treated tumor-bearing animals in order to address all safety concerns related to the plasticity of MSCs and their possible pro-tumorigenic effects.

## Figures and Tables

**Table 1:** Clinical studies using MSC-based therapies for cancer treatment.

Type of MSCs	Route of Injection	Tumor Type/Disease	Clinical Trial ID	Status
IFN-β-expressing MSCs	intraperitoneal	ovarian cancer	NCT02530047	Completed
AT-MSCs	submandibular	radiation-induced xerostomia in previous head and neck cancer patients	NCT02513238	Completed
MSCs + UC-HSCs	intra-osseous	hematological malignancies	NCT02181478	Completed
BM-MSCs infected with an oncolytic adenovirus, ICOVIR-5 (CELYVIR)	intravenous	metastatic and refractory tumors	NCT01844661	Completed
BM-MSCs	intravenous	ARDS in patients with malignancies	NCT02804945	Completed
MV-NIS-infected MSC	intraperitoneal	recurrent ovarian, primary peritoneal, fallopian tube cancer	NCT02068794	Recruiting
TRAIL-expressing MSCs + cisplatin/pemetrexed	intravenous	metastatic NSCLC	NCT03298763	Recruiting
BM-MSCs infected with an oncolytic adenovirus, ICOVIR-5 (CELYVIR)	intravenous	DIPG medulloblastoma	NCT04758533	Recruiting

## Data Availability

Not applicable.

## Abbreviations

ClinicalTrials.gov Identifier: (Clinical trial ID), interferon beta (IFN-β), measles virus encoding thyroidal sodium iodide symporter (MV-NIS); adipose tissue derived MSCs (AT-MSCs), umbilical cord derived hematopoietic stem cells (UC-HSCs); Non-small cell lung cancer (NSCLC); Tumor necrosis factor-related apoptosis-inducing ligand (TRAIL); bone marrow-derived MSCs (BM-MSCs); Diffuse Intrinsic Pontine Glioma (DIPG); Acute Respiratory Distress Syndrome (ARDS).

## References

[1] Wu Z., Li S., Zhu X. (2021). The Mechanism of Stimulating and Mobilizing the Immune System Enhancing the Anti-Tumor Immunity. Front. Immunol..

[2] Alfei F., Ho P.C., Lo W.L. (2021). DCision-making in tumors governs T cell anti-tumor immunity. Oncogene.

[3] Qiu Y., Chen T., Hu R., Zhu R., Li C., Ruan Y., Xie X., Li Y. (2021). Next frontier in tumor immunotherapy: Macrophage-mediated immune evasion. Biomark. Res..

[4] Kaschek L., Zöphel S., Knörck A., Hoth M. (2021). A calcium optimum for cytotoxic T lymphocyte and natural killer cell cytotoxicity. Semin. Cell Dev. Biol..

[5] Hamilton G., Plangger A. (2021). The Impact of NK Cell-Based Therapeutics for the Treatment of Lung Cancer for Biologics: Targets and Therapy. Biologics.

[6] Najafi M., Farhood B., Mortezaee K. (2019). Contribution of regulatory T cells to cancer: A review. J. Cell Physiol..

[7] Harrell C.R., Jankovic M.G., Fellabaum C., Volarevic A., Djonov V., Arsenijevic A., Volarevic V. (2019). Molecular Mechanisms Responsible for Anti-inflammatory and Immunosuppressive Effects of Mesenchymal Stem Cell-Derived Factors. Adv. Exp. Med. Biol..

[8] Harrell C.R., Djonov V., Volarevic V. (2021). The Cross-Talk between Mesenchymal Stem Cells and Immune Cells in Tissue Repair and Regeneration. Int. J. Mol. Sci..

[9] Yuan J., Wei Z., Xu X., Ocansey D.K.W., Cai X., Mao F. (2021). The Effects of Mesenchymal Stem Cell on Colorectal Cancer. Stem Cells Int..

[10] Weng Z., Zhang B., Wu C., Yu F., Han B., Li B., Li L. (2021). Therapeutic roles of mesenchymal stem cell-derived extracellular vesicles in cancer. J. Hematol. Oncol..

[11] Razeghian E., Margiana R., Chupradit S., Bokov D.O., Abdelbasset W.K., Marofi F., Shariatzadeh S., Tosan F., Jarahian M. (2021). Mesenchymal Stem/Stromal Cells as a Vehicle for Cytokine Delivery: An Emerging Approach for Tumor Immunotherapy. Front. Med..

[12] Hassanzadeh A., Altajer A.H., Rahman H.S., Saleh M.M., Bokov D.O., Abdelbasset W.K., Marofi F., Zamani M., Yaghoubi Y., Yazdanifar M. (2021). Mesenchymal Stem/Stromal Cell-Based Delivery: A Rapidly Evolving Strategy for Cancer Therapy. Front. Cell Dev. Biol..

[13] Sun Z., Zhang J., Li J., Li M., Ge J., Wu P., You B., Qian H. (2021). Roles of Mesenchymal Stem Cell-Derived Exosomes in Cancer Development and Targeted Therapy. Stem Cells Int..

[14] Ghosh T., Barik S., Bhuniya A., Dhar J., Dasgupta S., Ghosh S., Sarkar M., Guha I., Sarkar K., Chakrabarti P. (2016). Tumor-associated mesenchymal stem cells inhibit naïve T cell expansion by blocking cysteine export from dendritic cells. Int. J. Cancer.

[15] Srivastava M.K., Sinha P., Clements V.K., Rodriguez P., Ostrand-Rosenberg S. (2010). Myeloid-derived suppressor cells inhibit T-cell activation by depleting cystine and cysteine. Cancer Res..

[16] Jia X.H., Feng G.W., Wang Z., Du Y., Shen C., Hui H., Peng D., Li Z., Kong D.L., Tian J. (2016). Activation of mesenchymal stem cells by macrophages promotes tumor progression through immune suppressive effects. Oncotarget.

[17] Mathew E., Brannon A.L., Del Vecchio A., Garcia P.E., Penny M.K., Kane K.T., Vinta A., Buckanovich R.J., di Magliano M.P. (2016). Mesenchymal Stem Cells Promote Pancreatic Tumor Growth by Inducing Alternative Polarization of Macrophages. Neoplasia.

[18] de Lourdes Mora-García M., García-Rocha R., Morales-Ramírez O., Montesinos J.J., Weiss-Steider B., Hernández-Montes J., Ávila-Ibarra L.R., Don-López C.A., Velasco-Velázquez M.A., Gutiérrez-Serrano V. (2016). Mesenchymal stromal cells derived from cervical cancer produce high amounts of adenosine to suppress cytotoxic T lymphocyte functions. J. Transl. Med..

[19] Liu Z., Mi F., Han M., Tian M., Deng L., Meng N., Luo J., Fu R. (2021). Bone marrow-derived mesenchymal stem cells inhibit CD8^+^ T cell immune responses via PD-1/PD-L1 pathway in multiple myeloma. Clin. Exp. Immunol..

[20] Guo S., Huang C., Han F., Chen B., Ding Y., Zhao Y., Chen Z., Wen S., Wang M., Shen B. (2021). Gastric Cancer Mesenchymal Stem Cells Inhibit NK Cell Function through mTOR Signalling to Promote Tumour Growth. Stem Cells Int..

[21] Galland S., Vuille J., Martin P., Letovanec I., Caignard A., Fregni G., Stamenkovic I. (2017). Tumor-Derived Mesenchymal Stem Cells Use Distinct Mechanisms to Block the Activity of Natural Killer Cell Subsets. Cell Rep..

[22] Song J.H., Eum D.Y., Park S.Y., Jin Y.H., Shim J.W., Park S.J., Kim M.-Y., Park S.J., Heo K., Choi Y.J. (2020). Inhibitory effect of ginsenoside Rg3 on cancer stemness and mesenchymal transition in breast cancer via regulation of myeloid-derived suppressor cells. PLoS ONE..

[23] Sarhan D., Wang J., Arvindam U.S., Hallstrom C., Verneris M.R., Grzywacz B., Warlick E., Blazar B.R., Miller J.S. (2020). Mesenchymal stromal cells shape the MDS microenvironment by inducing suppressive monocytes that dampen NK cell function. JCI Insight.

[24] Vladimirovna I.L., Sosunova E., Nikolaev A., Nenasheva T. (2016). Mesenchymal Stem Cells and Myeloid Derived Suppressor Cells: Common Traits in Immune Regulation. J. Immunol. Res..

[25] Hatzioannou A., Boumpas A., Papadopoulou M., Papafragkos I., Varveri A., Alissafi T., Verginis P. (2021). Regulatory T Cells in Autoimmunity and Cancer: A Duplicitous Lifestyle. Front. Immunol..

[26] Ling W., Zhang J., Yuan Z., Ren G., Zhang L., Chen X., Rabson A.B., Roberts A.I., Wang Y., Shi Y. (2014). Mesenchymal stem cells use IDO to regulate immunity in tumor microenvironment. Cancer Res..

[27] Heidari F., Razmkhah M., Razban V., Erfani N. (2021). Effects of indoleamine 2,3-dioxygenase (IDO) silencing on immunomodulatory function and cancer-promoting characteristic of adipose-derived mesenchymal stem cells (ASCs). Cell Biol. Int..

[28] Mehdipour F., Razmkhah M., Rezaeifard S., Bagheri M., Talei A.R., Khalatbari B., Ghaderi A. (2018). Mesenchymal stem cells induced anti-inflammatory features in B cells from breast tumor draining lymph nodes. Cell Biol. Int..

[29] Gazdic M., Markovic B.S., Jovicic N., Misirkic-Marjanovic M., Djonov V., Jakovljevic V., Arsenijevic N., Lukic M., Volarevic V. (2017). Mesenchymal Stem Cells Promote Metastasis of Lung Cancer Cells by Downregulating Systemic Antitumor Immune Response. Stem Cells Int..

[30] Gazdic M., Volarevic V., Arsenijevic N., Stojkovic M. (2015). Mesenchymal stem cells: A friend or foe in immune-mediated diseases. Stem Cell Rev. Rep..

[31] Davies L.C., Heldring N., Kadri N., Le Blanc K. (2017). Mesenchymal Stromal Cell Secretion of Programmed Death-1 Ligands Regulates T Cell Mediated Immunosuppression. Stem Cells.

[32] Miloradovic D., Miloradovic D., Markovic B.S., Acovic A., Harrell C.R., Djonov V., Arsenijevic N., Volarevic V. (2020). The Effects of Mesenchymal Stem Cells on Antimelanoma Immunity Depend on the Timing of Their Administration. Stem Cells Int..

[33] Volarevic V., Ljujic B., Stojkovic P., Lukic A., Arsenijevic N., Stojkovic M. (2011). Human stem cell research and regenerative medicine—Present and future. Br. Med. Bull..

[34] Szoor A., Vaidya A., Velasquez M.P., Mei Z., Galvan D.L., Torres D., Gee A., Heczey A., Gottschalk S. (2017). T Cell-Activating Mesenchymal Stem Cells as a Biotherapeutic for HCC. Mol. Ther. Oncolytics.

[35] Magistri P., Leonard S.Y., Tang C.M., Chan J.C., Lee T.E., Sicklick J.K. (2014). The glypican 3 hepatocellular carcinoma marker regulates human hepatic stellate cells via Hedgehog signaling. J. Surg. Res..

[36] Feng H., Zhao J.K., Schiergens T.S., Wang P.X., Ou B.C., Al-Sayegh R., Li M.L., Lu A.G., Yin S., Thasler W.E. (2018). Bone marrow-derived mesenchymal stromal cells promote colorectal cancer cell death under low-dose irradiation. Br. J. Cancer.

[37] Harrell C.R., Jovicic N., Djonov V., Volarevic V. (2020). Therapeutic Use of Mesenchymal Stem Cell-Derived Exosomes: From Basic Science to Clinics. Pharmaceutics.

[38] Harrell C.R., Jovicic N., Djonov V., Arsenijevic N., Volarevic V. (2019). Mesenchymal Stem Cell-Derived Exosomes and Other Extracellular Vesicles as New Remedies in the Therapy of Inflammatory Diseases. Cells.

[39] Xu Y., Shen L., Li F., Yang J., Wan X., Ouyang M. (2019). microRNA-16-5p-containing exosomes derived from bone marrow-derived mesenchymal stem cells inhibit proliferation, migration, and invasion, while promoting apoptosis of colorectal cancer cells by downregulating ITGA2. J. Cell Physiol..

[40] Li T., Wan Y., Su Z., Li J., Han M., Zhou C. (2021). Mesenchymal Stem Cell-Derived Exosomal microRNA-3940-5p Inhibits Colorectal Cancer Metastasis by Targeting Integrin α6. Dig. Dis. Sci..

[41] Chen H.L., Li J.J., Jiang F., Shi W.J., Chang G.Y. (2020). MicroRNA-4461 derived from bone marrow mesenchymal stem cell exosomes inhibits tumorigenesis by downregulating COPB2 expression in colorectal cancer. Biosci. Biotechnol. Biochem..

[42] Liu L., Yu T., Jin Y., Mai W., Zhou J., Zhao C. (2021). MicroRNA-15a Carried by Mesenchymal Stem Cell-Derived Extracellular Vesicles Inhibits the Immune Evasion of Colorectal Cancer Cells by Regulating the KDM4B/HOXC4/PD-L1 Axis. Front. Cell Dev. Biol..

[43] Pakravan K., Babashah S., Sadeghizadeh M., Mowla S.J., Mossahebi-Mohammadi M., Ataei F., Dana N., Javan M. (2017). MicroRNA-100 shuttled by mesenchymal stem cell-derived exosomes suppresses in vitro angiogenesis through modulating the mTOR/HIF-1α/VEGF signaling axis in breast cancer cells. Cell Oncol..

[44] Babajani A., Soltani P., Jamshidi E., Farjoo M.H., Niknejad H. (2020). Recent Advances on Drug-Loaded Mesenchymal Stem Cells with Anti-neoplastic Agents for Targeted Treatment of Cancer. Front. Bioeng. Biotechnol..

[45] Layek B., Sadhukha T., Prabha S. (2016). Glycoengineered mesenchymal stem cells as an enabling platform for two-step targeting of solid tumors. Biomaterials.

[46] Layek B., Sadhukha T., Panyam J., Prabha S. (2018). Nano-Engineered Mesenchymal Stem Cells Increase Therapeutic Efficacy of Anticancer Drug Through True Active Tumor Targeting. Mol. Cancer Ther..

[47] Layek B., Shetty M., Nethi S.K., Sehgal D., Starr T.K., Prabha S. (2020). Mesenchymal Stem Cells as Guideposts for Nanoparticle-Mediated Targeted Drug Delivery in Ovarian Cancer. Cancers.

